# Trypan blue removal from water with zein sorbents and laccase

**DOI:** 10.1007/s42452-020-04107-w

**Published:** 2021-01-07

**Authors:** Tatianna Marshall, Kristine Lamont, Alejandro G. Marangoni, Loong-Tak Lim, Xiuju Wang, Erica Pensini

**Affiliations:** 1 School of Engineering, University of Guelph, 50 Stone Road East, Guelph, ON N1G 2W1 Canada; 2 Food Science Department, University of Guelph, 50 Stone Road East, Guelph, ON N1G 2W1 Canada

**Keywords:** Sorption, Dye, Trypan blue, Water treatment, Zein, Laccase

## Abstract

**Abstract:**

Zein-based materials were used to remove Trypan blue from water under flow conditions and in batch tests. In flow tests, zein dissolved at pH = 13 was injected in sand columns and subsequently coagulated with CaCl_2_, to create an adsorbent filter which removed over 99% of Trypan blue. Batch tests were conducted using zein powder, zein dissolved at pH = 13 and coagulated with CaCl_2_, Fe_2_Cl_3_ or citric acid, and zein dissolved in ethanol and then coagulated with water. The highest Trypan blue removal was achieved with zein powder (4000 mg Trypan blue/kg sorbent, as determined through spectrophotometry), followed by zein coagulated with Fe_2_Cl_3_ (500 mg Trypan blue/kg sorbent) and with other salts (140 mg Trypan blue/kg sorbent). Differences in the sorption efficiency are attributed to differences in the surface area. The sorption isotherm of Trypan blue onto zein-based sorbents was a Type II isotherm, suggesting physisorption. Desorption of Trypan blue was limited when zein-based coagulated sorbents were immersed in pure water. Trypan blue could be degraded by free laccase in water, as determined through spectrophotometry and electrospray ionization mass spectroscopy (ESI-MS). Trypan blue could also be degraded by laccase when zein-based laccase-containing sorbents were prepared at pH = 10, using Fe_2_Cl_3_ as coagulant.

**Graphic abstract:**

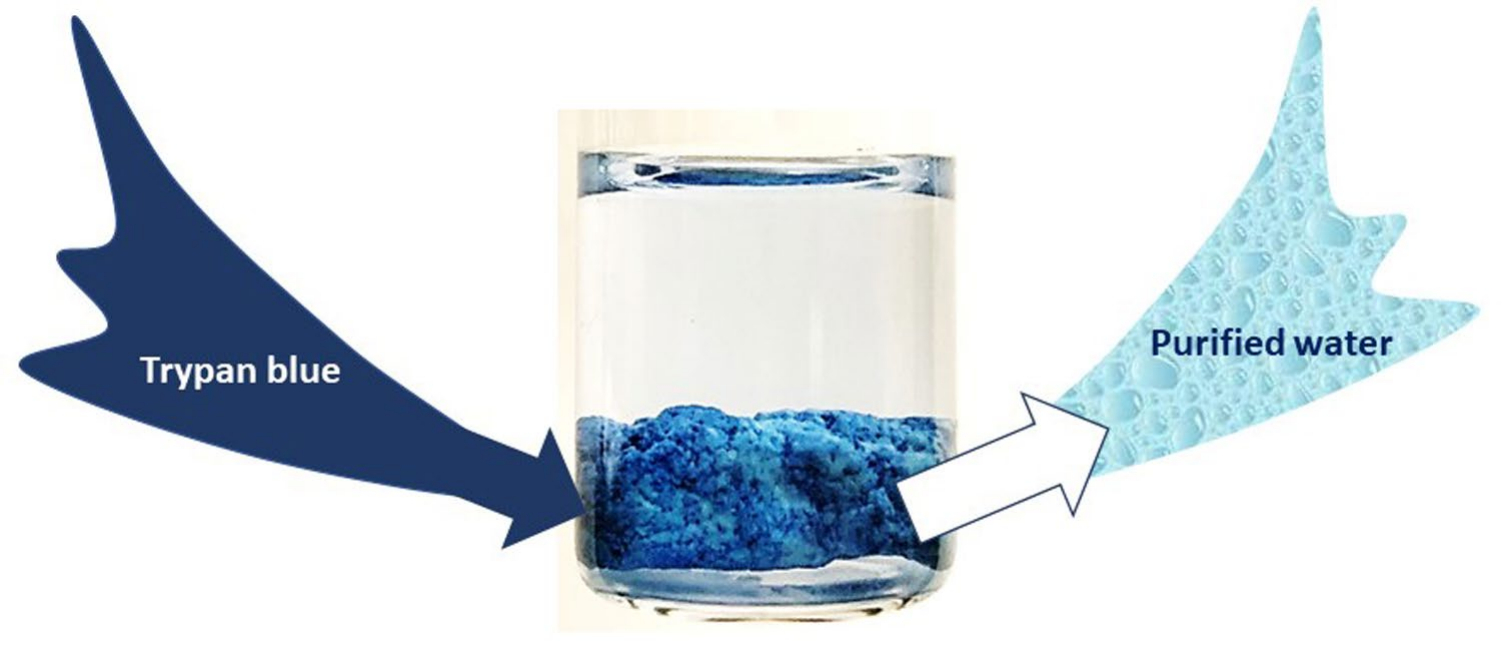

**Supplementary information:**

The online version contains supplementary material available at 10.1007/s42452-020-04107-w.

## Introduction

Pollution from industrial processes has recently become a significant worldwide concern [1]. The range of pollutants released into the environment is broad and includes dyes. Dyes are used in the textile industry [2] and in other industries [3], including paper printing, color photography, pharmaceutical, food, cosmetic, and leather industries [4]. Despite their widespread use, dyes are toxic to humans and to the environment. Studies have for instance demonstrated the toxicity of azo dyes, anthraquinone and other dyes [5, 6]. Trypan blue is an azo dye used for biological staining [7], and its toxicity is known. Trypan blue is a teratogen [8], and it can harm the human eye [9]. In addition to their toxicity, when dyes are released in water bodies, they reduce light penetration in water, and they prevent oxygen ingress and increase biochemical oxygen demand [4]. Since dyes are clearly detectable, the aesthetic damage is also of concern [4]. Although industrial effluents should receive treatment prior to discharge, dyes are still being released in the environment [4]. Azo dyes represent approximately half of all dyes used, and they are the largest group of synthetic colorants released into the environment [4].

Previous studies successfully removed different dyes with reverse osmosis [10], with coagulants and flocculants [11], oxidizing agents and Fenton’s reagents [12], ozonation and electrochemical methods [13], photocatalysis [14], sonolysis [15], biological methods using fungi and aerobic and anaerobic bacteria [16], sorption and ionic exchange [17].

Different sorbents have been proposed for the removal of dyes from water, including sulfuric acid treated sawdust [18], apple pomace and wheat straw [19], immobilized fungi [20], activated carbon, bagasse, husk, date pits, corncob, pinewood and pith [21], and sunflower stalks (used to remove direct blue dye) [16]. Nanoparticles have also been used for the removal of dyes, including TiO_2_ nanoparticles (which have been investigated for their sorption capacity and photocatalytic efficiency [22]) and hollow zein nanoparticles for the removal of reactive blue [23]. Polyvinyl alcohol membranes containing scleroglucan, cellulose microfibers, or zein have been used for the removal of crystal violet [24]. Zein is a degradable, inexpensive, by-product of corn, and it is therefore an attractive material for environmental applications [25]. Moreover, white rot fungi, which produce the enzyme laccase, can effectively decolorize dye wastewater [26]. Enzymes, and specifically the fungal enzyme laccase, have been used to decolorize and detoxify water polluted with different types of dyes [27–29], including Trypan blue [30]. Laccases are copper-containing oxidoreductases, which can complete one electron oxidation of diverse environmental contaminants [31], including phenolic compounds such as pesticides [32] and furan aldehydes [33], in addition to dyes. Enzymes used for decolorization can be either dispersed in water or immobilized, for instance on modified sand [27]. While both fungal biomass and free laccase can remove Trypan blue from water, a thermokinetic study found that decolorization reactions were spontaneous in nature over a wider temperature range for free laccase than for those with fungal biomass [34]. These results demonstrate the ability of free laccase to withstand large fluctuations in temperature without affecting reaction spontaneity [34]. Some studies have used immobilized laccase to treat dyes or other contaminants. Immobilization scaffolds include chitosan-based materials [35], polyamide/chitosan nanofibers [36], and zein/polyurethane materials [32]. Laccase was also immobilized on zein fibers, used to produce temperature indicators [37]. Laccase immobilization on chitosan and polyamide/chitosan nanofibers was achieved using glutaraldehyde [35, 36].

While Reactive Blue 19 has been sorbed onto nylon–zein fibers [38], zein was not used before for the removal of Trypan blue from water. The effectiveness of laccase in degrading Trypan blue pre-sorbed onto zein sorbents was also not investigated in previous studies. Our study addresses the current gaps by investigating removal of Trypan blue from water using zein sorbents under no-flow and continuous stirring conditions (batch tests), and by analyzing the effectiveness of laccase in degrading Trypan blue sorbed onto zein sorbents. These conditions are for instance found in industrial treatment facilities or in engineered water collection ponds. Finally, our study investigates the removal of dyes from water under flow conditions, using a sand column mimicking sandy aquifers. In flow tests, zein sorbent materials were obtained by injecting dissolved zein in a soil column, and subsequently solidifying it, to create a filtering medium. These injectable filtering materials can be located around water bodies such as contaminated lakes, rivers or canals. These filters can protect aquifers recharged from the surface water bodies, by cleaning surface waters flowing into the subsurface.

## Materials and methods

### Materials

Zein (purified), NaOH (pellets, 95–100%, SpectrumTM), CaCl_2_ · 2H_2_O (reagent grade), Fe_2_Cl_3_ · 2H_2_O (reagent grade), citric acid (anhydrous, food grade), Trypan blue (ACROS organics), and ethanol anhydrous (EtOH) (95%) were purchased from Fisher Scientific (Canada). Laccase (from *Trametes versicolor*) was purchased from Sigma-Aldrich. Antiskid sand (Charbonneau Floral Ltd., Canada) was purchased from a local market. Deionized (DI) water was utilized in all tests conducted.

### Preparation of zein sorbents for batch tests with Trypan blue

Zein sorbents used for Trypan blue removal were prepared using four methods. In the first method, they were prepared by solubilizing 33 g/L of zein in DI water at pH = 13 and then coagulating it using different coagulants: (1) CaCl_2_; (2) Fe_2_Cl_3_; (3) citric acid. Different coagulants were tested because they could potentially affect the electrostatic charge of the sorbents and hence their effectiveness in removing Trypan blue. The final concentrations of CaCl_2_ and Fe_2_Cl_3_ were 0.07 M (and stock solutions had 2 M concentrations). Stock solutions of citric acid had a concentration of 50% (weight based) and were added to zein solutions to obtain a final citric acid concentration of 33 g/L of water. In the second method, laccase (7·10^–2^ g/L) was incorporated in part of the zein samples coagulated with CaCl_2_, by adding it to zein solutions at pH = 13 before the coagulation step with CaCl_2_. In the third method zein sorbents were prepared using 15 g/L of zein, first dissolved at pH = 13, before lowering the pH to pH = 10. Laccase was subsequently added at 0.35 g/L, before solidifying the sorbents with Fe_2_Cl_3_ (to obtain a 0.1 M Fe_2_Cl_3_). This method was used in our previous study [39]. In the fourth method zein was dissolved in ethanol (200 g/L) and coagulated by adding DI water (to obtain a 20% ethanol, 80% water mixture). When using these concentrations, zein-based materials formed a cohesive mass, and will be referred to as “coagulated.” Zein sorbents prepared with these four methods were rinsed with DI water and used for Trypan blue removal in batch tests immediately after preparation. Zein powder was also used without further treatment for the removal of Trypan blue from water. In all instances, the pH was adjusted with NaOH and HCl.

### Electrospray ionization mass spectrometry (ESI-MS)

Trypan blue samples treated with either free laccase in DI water (38 mg/L of Trypan blue and 250 mg/L laccase) or laccase in 0.5 M Fe_2_Cl_3_ solutions (38 mg/L of Trypan blue) were analyzed using a Thermo LCQ Fleet Ultimate 3000 LC-MS and Xcalibur software. Tests conducted with Fe_2_Cl_3_ solution were aimed at understanding the effect of iron ions on Trypan blue degradation by laccase, because Fe_2_Cl_3_ was used as a coagulant for zein sorbents (Sect. 2.2). The 0.5 M Fe_2_Cl_3_ solution concentration was selected because it was well above the concentration used to make zein sorbents (0.07 M), and it would hence allow clearly noticing any effect of iron on Trypan blue degradation. Samples were directly analyzed by ESI-MS, bypassing the liquid chromatography column. Trypan blue samples without laccase or Fe_2_Cl_3_ were also analyzed for comparison. All samples were diluted with DI water and analyzed at pH = 11 (adjusted with NaOH). This pH was used to ensure that laccase could remain active when sorbents were prepared at alkaline pH.

### Trypan blue removal from water in batch tests: sorption, desorption and enzymatic degradation

Batch tests were conducted at room temperature (21 °C) and at pH = 6 to test the kinetics of Trypan blue removal from water with either free laccase or with zein sorbents. In tests conducted with free laccase, laccase (125 mg/L) was added to Trypan blue solutions in DI water (19 mg/L). Tests were also conducted using zein sorbents with and without laccase, by immersing the sorbents in Trypan blue solutions under either quiescent conditions or continuous mixing (using a magnetic stir bar). Trypan blue concentrations were then monitored over time for a total of either 96 h (4 days, under quiescent conditions) or 24 h (under continuous mixing conditions), and determined using a Hach spectrophotometer at 605 nm wavelength. A separate set of batch tests were conducted at 21 °C to determine the sorption isotherms with zein-based sorbents coagulated with CaCl_2_ under either quiescent conditions or continuous mixing. Desorption tests were conducted by immersing zein sorbents used in 24 h sorption tests in DI water, and analyzing Trypan blue concentrations after 24 h under either quiescent conditions or continuous mixing.

### Trypan blue filtration through zein sorbents injected into sand (flow tests)

Zein (33 g/L) was dissolved in DI water at pH = 13. Zein solution (5 mL) was injected on a sand column (6 mL), obtained using a Falcon tube pierced at the bottom. Zein was then solidified by injecting 0.5 mL of 2 M solution of CaCl_2_ · 2H_2_O on top of the column. The column was then flushed with 2 mL of 19 mg/L Trypan blue solution, without collecting water samples exiting the sand column (because they contained zein residuals). Trypan blue (19 mg/L) was then added to the column again and the water samples were collected at the outlet of the sand column, to determine the reduction in Trypan blue concentration. This process was repeated six times, using 3 mL of Trypan blue solution each time to the column, and analyzing the concentration reduction after each addition. Each water sample was analyzed using a Hach spectrophotometer, at a 605 nm wavelength.

### Zeta potential measurements

Zeta potential measurements were conducted at 23 °C with a Malvern zetasizer on zein particles formed by adding CaCl_2_ (7 × 10^–2^ M), Fe_2_Cl_3_ (10^–2^ M) and citric acid (33 g/L of water) to 200 mg/L zein solutions at pH = 13 (adjusted with NaOH). The concentration of Fe_2_Cl_3_ was kept low to control the formation of large flocs at basic pH. Particles were also produced by adding 20 mg of zein in 1 mL of ethanol, and subsequently diluting with 200 mL using DI water. The zeta potential of particles prepared with this method should be similar to the zeta potential of zein sorbent materials (which cannot be analyzed using a zetasizer due to their large size). Measurements were conducted with and without Trypan blue in solution (0.7 mg/L), to gain insights regarding the effect of electrostatic forces on Trypan blue sorption onto zein. The refractive index used was 1.45 for zein particles and 1.33 for water, as previously described [25, 40].

## Results and discussion

**Fig. 1:**
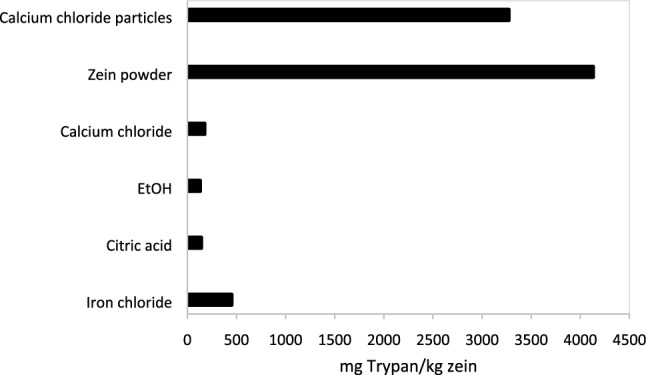
Trypan blue sorption onto zein sorbents after 24 h sorption under quiescent conditions, as determined using spectrophotometry. The initial Trypan blue concentration in water was 19 mg/L. The standard deviation between measurements was below 1 mg Trypan/kg zein

**Fig. 2:**
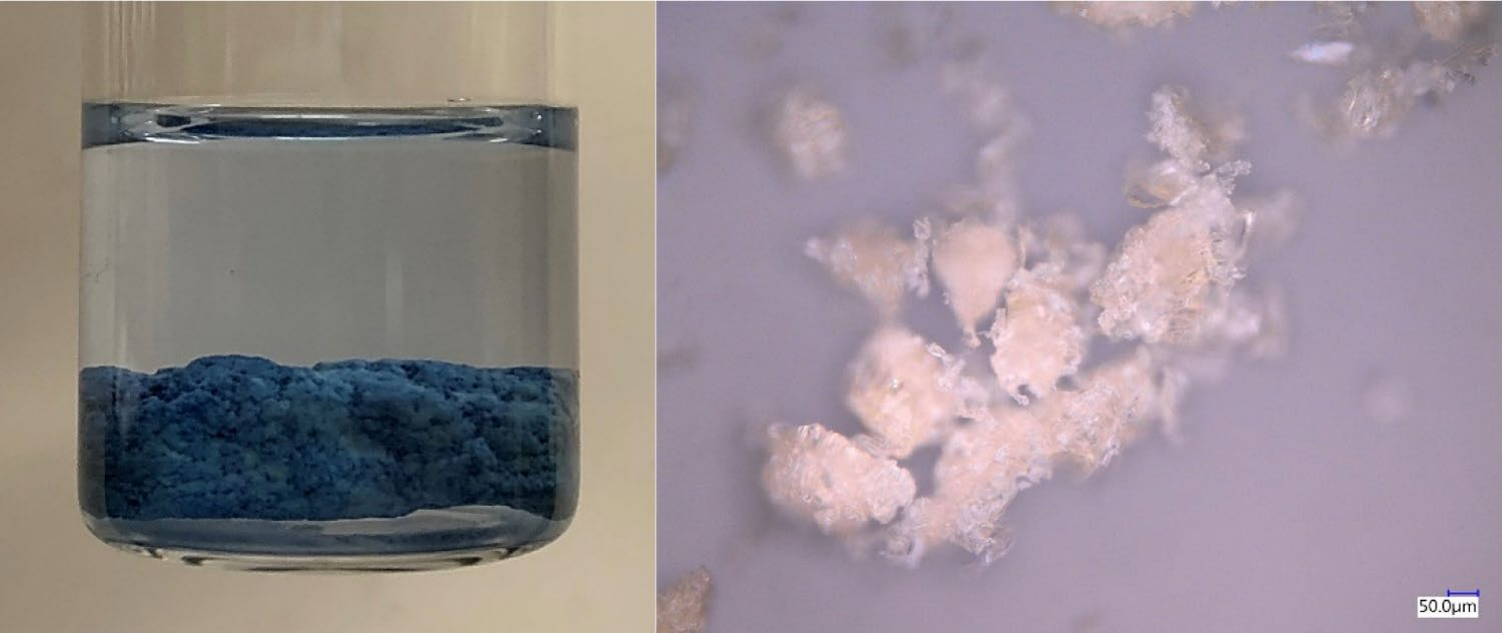
Example of compacted zein-based sorbent prepared with zein and CaCl_2_ (left) and optical microscopy image of zein powder (right)

Trypan blue was sorbed with either zein powder, or with coagulated zein-based sorbents, without laccase. Coagulation of zein with salts was previously reported [25]. Removal efficiencies of zein sorbents in sorbing Trypan blue from water in batch tests conducted under quiescent conditions over 24 h were determined using spectrophotometry and a calibration curve (Supporting Information, Fig. SI.1) and are given in Fig. 1. Sorption was most significant with zein powder, likely due to its high surface area compared to zein-based sorbents coagulated with CaCl_2_, Fe_2_Cl_3_ or citric acid, or obtained by adding water to zein solutions in ethanol (Figure 2). While more effective in removing Trypan blue, zein powder may be more challenging to recover from water than coagulated zein-based sorbents. Future research should focus on increasing the surface area of zein-based sorbents, while ensuring that they can be easily removed from water after treatment or used under flow conditions.

The coagulant used to prepare lumped zein sorbents affected their sorption efficiency, which increased in the following order: Fe_2_Cl_3_ > CaCl_2_ $\cong$ citric acid $\cong$ EtOH (Fig. 1). Zeta potential measurements were conducted on zein prepared at low zein concentrations, in the native solutions in which they were prepared, to gain insights regarding the effect of electrostatic interactions on Trypan blue sorption onto zein-based sorbents. Zeta potential measurements showed that zein particles obtained by adding CaCl_2_ to zein solution at pH = 13 were approximately neutral, in agreement with our previous study [25]. It is likely that coagulated zein-based sorbents were positively charged at pH = 6, at which sorption experiments were conducted after rinsing coagulated zein sorbents. At pH = 13, the zeta potential of zein particles obtained with Fe_2_Cl_3_ was $\cong$ − 16 mV (pH $\cong$ 12), whereas it was $\cong$ +11 mV and $\cong$ +18 mV when particles were prepared with citric acid (pH $\cong$ 3) and EtOH (pH $\cong$ 6), respectively. Iron likely dissociated and then formed iron oxides and hydroxides in water, the point of zero charge of which is at approximately pH = 8–10 [41]. Above the point of zero charge, iron present in the zein particles was likely responsible for their negative electric charge, whereas at pH = 6 iron oxides should be positively charged. A positive zeta potential was previously reported for zein particles produced using ethanol and water, and attributed to amino acid ionization [42]. Finally, the low pH measured with citric acid may account for the positive zeta potential of zein particles. Trypan blue is negatively charged, due to the hydroxyl groups and the SO_3_^−^ groups. Electrostatic interactions may have contributed to physisorption of Trypan blue onto zein sorbents at pH = 6. The effectiveness of zein-based sorbents coagulated with Fe_2_Cl_3_ suggests a specific affinity of Trypan blue for iron. Sorption and reduction of azo dyes on iron and iron-containing materials was previously reported. For instance, nanoscale zero-valent iron particles were used to remove the azo dye AB24 from water through reduction at pH = 9 [43]. The azo dye Congo red was sorbed onto iron composite nanoparticles [44]. Other azo dyes, both anionic and cationic, including Direct Blue 71, Acid Blue 40 and Basic Violet 16, were sorbed onto iron-based waterworks sludge [45]. These published results suggest that electrostatic interactions did not control sorption of dye onto iron sorbents coagulated with Fe_2_Cl_3_, since sorption onto iron compounds was previously observed for both anionic and cationic azo dyes. While more effective in sorbing Trypan blue, lumped zein sorbents coagulated with Fe_2_Cl_3_ released small quantities of iron, as determined with the naked eye based on the discoloration of water. Therefore, the use of CaCl_2_ could be preferred to Fe_2_Cl_3_ for zein coagulation. It is also speculated that hydrophobic interactions between the hydrophobic portions of zein and the aromatic groups of Trypan blue contributed to sorption. Some authors hypothesized hydrogen bonding between selected azo dyes onto zein (rather than physisorption). For instance, some studies suggested hydrogen bonding between the OH and NH_2_ groups of the azo dye Reactive Blue 19 dye and the N–H groups of zein [23], and hydrogen bonding has also been hypothesized between reactive black dye onto zein nanofibers [46].

**Fig. 3:**
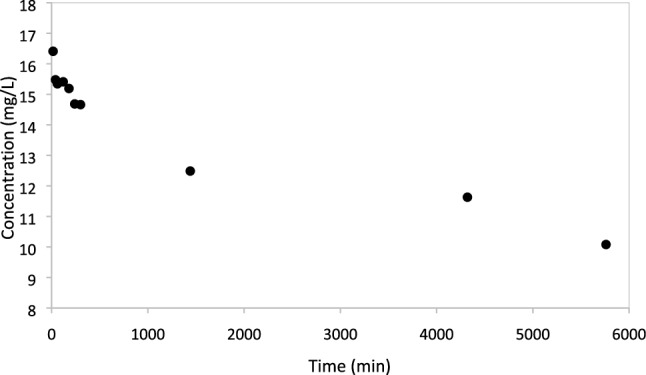
Sorption of Trypan blue (19 mg/L) onto zein coagulated with CaCl_2_ over time under quiescent conditions. The sorbent mass used was 0.1 kg/L

Sorption of Trypan blue onto zein sorbents coagulated with CaCl_2_ occurred gradually under quiescent conditions, as shown in Fig. 3. The kinetics of Trypan blue removal could have been due to either mass transfer limitations or to the sorption mechanisms. Experiments under stirring conditions were conducted to assess which factor controlled the kinetics of Trypan blue removal. Under stirring conditions, the concentration of Trypan blue decreased more rapidly, and was reduced from 19 to 12 mg/L after 300 min and finally to approximately 10 mg/L after 24 h. These results indicate that mass transfer could be promoted by mixing, improving the Trypan blue removal efficiency. Nonetheless, both under quiescent and continuous mixing conditions, sorption was not instantaneous. Chemisorption generally occurs instantaneously [47]. Therefore, the data suggest that hydrophobic interactions (rather than stronger bonds such as hydrogen bonds) controlled sorption of Trypan blue onto zein sorbents coagulated with CaCl_2_.

**Fig. 4:**
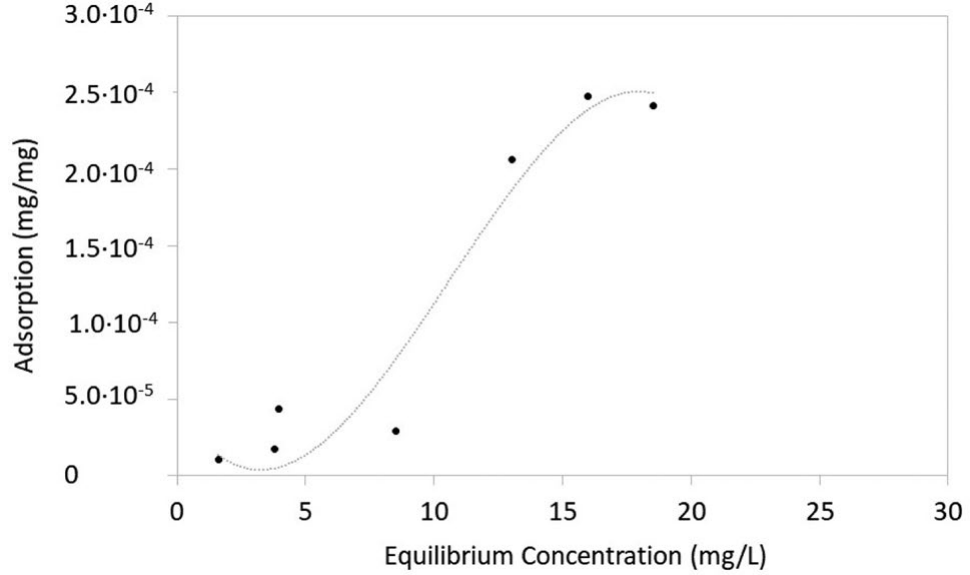
Sorption isotherm of Trypan blue onto zein coagulated with CaCl_2_. The curves were measured after 24 h sorption, under continuous stirring conditions

The sorption isotherm measured for Trypan blue sorption onto zein sorbents coagulated with CaCl_2_ after 24 h under stirring conditions had the characteristic s shape of a Type II sorption isotherm (Fig. 4). Type II isotherms are observed exclusively for physisorption [48], supporting the hypothesis of hydrophobic interactions rather than stronger chemical or hydrogen bonding between zein Trypan blue. It is noted that after 24 h sorption was lower without stirring. This result confirms that stirring promoted mass transfer and hence Trypan blue removal (as discussed above). Sorption after 24 h under quiescent conditions is shown in Fig. SI. 2 (Supporting Information file).

**Fig. 5:**
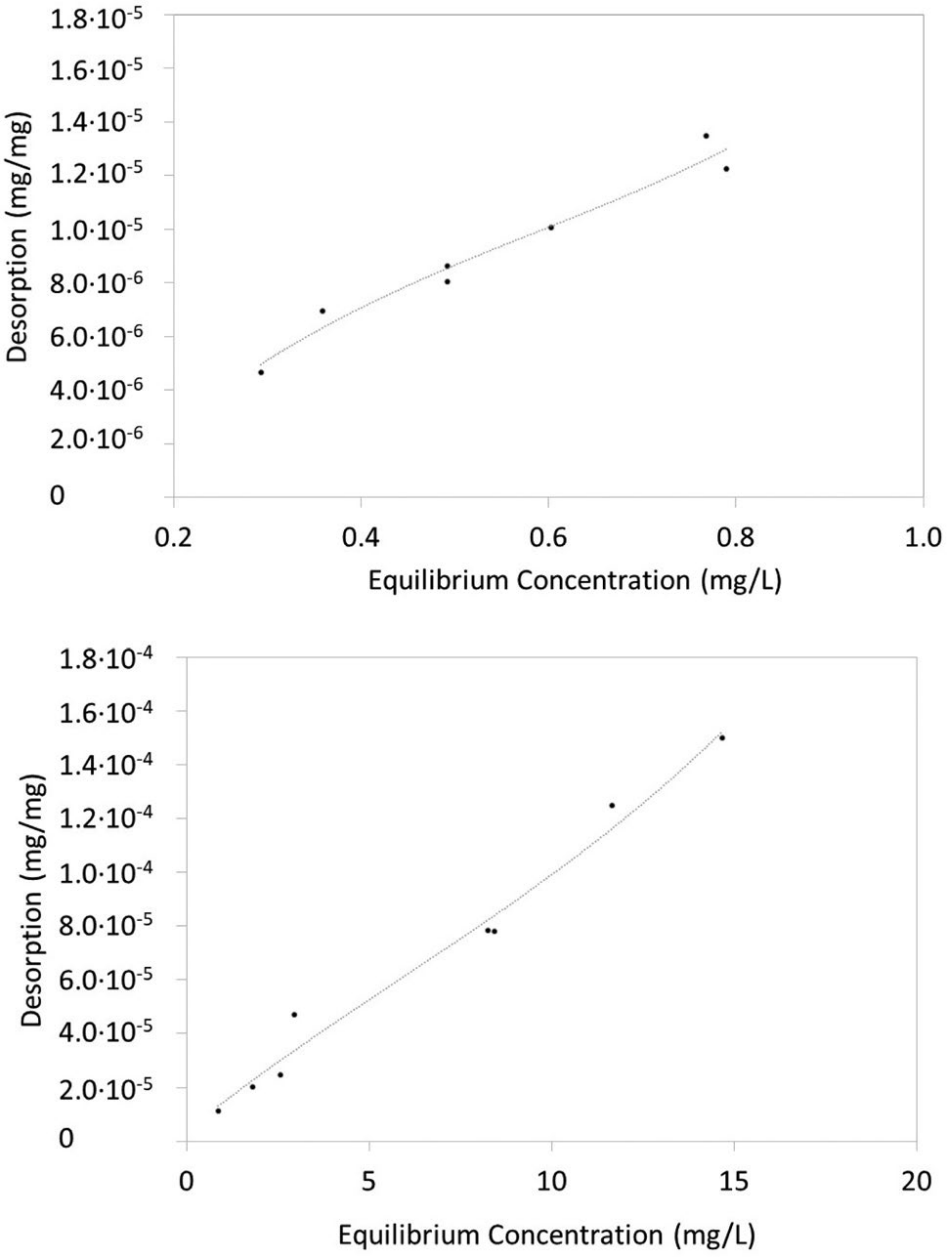
Desorption isotherm of Trypan blue from zein coagulated with CaCl_2_. Sorbents were immersed in DI water following 24 h sorption and equilibrated for an additional 24 h in DI water under quiescent conditions (top) or under mixing conditions (bottom)

Physisorption was confirmed by the fact that Trypan blue could desorb from zein-based sorbents coagulated with CaCl_2_ upon immersion in DI water under either quiescent or continuous stirring conditions, although desorption was limited over a period of 24 h (Fig. 5). It is noted that the sorption isotherm was obtained using Trypan blue concentrations in a range from approximately 1–38 mg/L. Investigating larger concentration ranges should be the objective of future research.

Finally, Trypan blue removal from water under flow conditions was tested, using zein filters obtained by injecting zein solutions in sand columns and subsequently coagulating them with CaCl_2_. After initially passing an aliquot of 3 mL through the zein filters, the concentration of Trypan blue decreased by over 99.5% (from a starting concentration of 19 mg/L). When additional aliquots of Trypan blue solutions were injected (3 mL each, up to a total of 6 aliquots), the concentration of Trypan blue decreased by 94.5% or more for each aliquot. Zein filters produced with a single injection of zein reduced flow by approximately 63%. Our previous study demonstrated that layered injection of zein could create barriers that reduced flow by ≈ 96% [25]. Here, the goal is not however to fully impede flow. It is envisioned that zein filters could be injected around surface water bodies contaminated by Trypan blue dye, allowing aquifer recharge with decontaminated water.

Previous studies successfully removed different dyes with diverse methods, including sorption and ionic exchange [17]. However, sorbents previously developed could not be produced in situ, for instance around river beds or lakes polluted with dyes. This is the first study in which sorbents for dye removal were produced in situ (at the lab scale) by injection of zein solutions inside a geological medium, followed by zein solidification using salt solutions.

It is noted that typical textile wastewater contains impurities such as acids, alkalis, salts, and metal ions (ex. Na^+^, K^+^, Cu^2+^, Ca^2+^, Cr^3+^) [49]. The presence of these impurities, especially cations, can increase the ionic strength, significantly impacting the adsorption of dyes [50]. Future work should then investigate the effect of wastewater impurities on Trypan blue sorption onto zein filters.

**Fig. 6:**
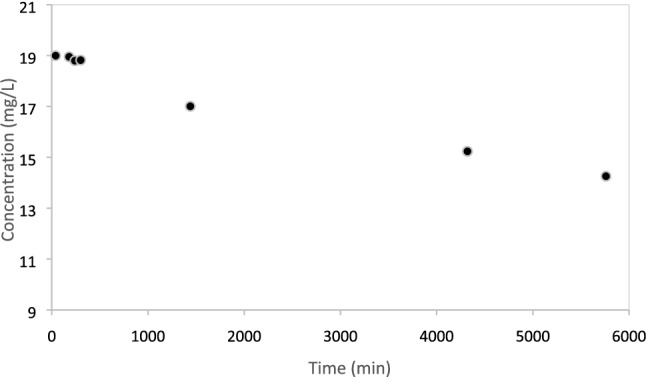
Concentration of Trypan blue in water, following degradation with 125 mg/L of laccase at neutral pH. The initial concentration of Trypan blue in water was 19 mg/L

While sorbents can remove Trypan blue from water, they do not degrade it. Laccase progressively reduced Trypan blue concentrations in water at neutral pH, as determined with spectrophotometry (Fig. 6). Trypan blue reduction was 25% in 96 h. Trypan blue degradation was also evident based on observations with the naked eye, which showed first a transition from blue to purple and then a decrease in the color intensity.

Previous studies successfully used free laccase to decolorize Trypan blue solutions [30, 34, 51]. The laccase-mediated degradation of azo dyes is attributed to a two-step process, in which one electron is abstracted from the phenolic/naphtholic ring to form a phenoxy radical, followed by the abstraction of a second electron to form an aromatic cation stabilized by electron-donating groups in the ring [30]. ESI-MS analyses indicated that laccase could also degrade Trypan blue at alkaline pH (pH = 11), in either DI water or in 0.5 M Fe_2_Cl_3_ solutions, as evident from the appearance of new peaks after either treatment for 24 h (Figs. SI.3–SI.5, Supporting Information). Possible characteristic peaks for Trypan blue at pH = 11 were identified at 295, 335, 401, 441, and 454 m/z. After treatment with laccase, the Trypan blue characteristic peaks remained with the addition of new major peaks at 507, 547, 613, 719, 825, and 931 m/z. The clear appearance of new m/z peaks suggests degradation of Trypan blue. After treatment with laccase in Fe_2_Cl_3_, solutions, the Trypan blue characteristic peaks again remained, along with identical m/z values for newly introduced peaks. Identical m/z values of Trypan blue with laccase and Trypan blue in Fe_2_Cl_3_ solutions could propose that possible degradation pathways are similar under both conditions.

Trypan blue degradation by laccase at alkaline pH and in the presence of iron chloride indicates that it would be possible to incorporate laccase in zein-based sorbents coagulated with Fe_2_Cl_3_ and prepared at alkaline pH to degrade Trypan blue (as will be discussed shortly here below). It is however noted that laccase activity is pH dependent, as we determined in our previous study [39]. This study demonstrated that free laccase activity was optimal at pH ≈5, but it remained active up to pH ≈ 10–11. This study also showed that iron promoted binding of laccase onto zein and its incorporation into zein sorbents coagulated with Fe_2_Cl_3_ [39]. Importantly, this study also shows that laccase retained its activity when it was incorporated inside zein sorbents coagulated with Fe_2_Cl_3_ and prepared at pH = 10 [39].

**Fig. 7:**
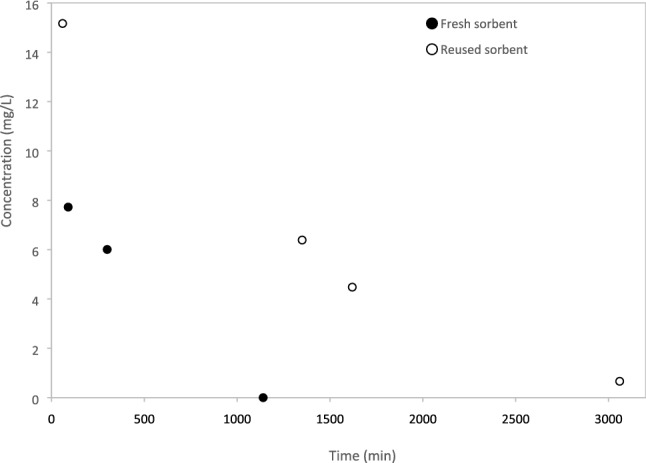
Removal of Trypan blue with zein sorbents coagulated with Fe_2_Cl_3_ with laccase (0.12 g laccase/g sorbent). The sorbent mass used was 37 g/L, and the initial Trypan blue concentration was 19 mg/L

Incorporating laccase inside zein sorbents is advantageous. Currently, methods have been developed to extract laccase at a lower cost compared to the past [52, 53]. Nonetheless, embedding laccase in zein sorbents would allow its reuse, hence further decreasing costs. Other methods of removal of dyes could also be relatively inexpensive, such as Fenton’s reagents [54] and activated carbon [55]. The advantage of using laccase-containing zein sorbents is that they would allow degradation (dissimilar to materials that can exclusively sorb), while also avoiding the use of chemicals. Laccase was therefore embedded into zein sorbents coagulated with Fe_2_Cl_3_ at pH = 10, with the goal of degrading Trypan blue following its sorption onto zein. The purple discoloration of the water indicated Trypan blue degradation, confirming that laccase remained active. Without laccase (sorption only), Trypan blue concentrations (and hence the blue color intensity) decreased (as described above), but the color did not change to purple. The surface of the zein sorbents in which laccase was embedded also turned purple upon exposure to Trypan blue (Fig. SI.6, Supporting Information). Importantly, the Trypan concentrations in water decreased more significantly when using sorbents containing laccase, even after sorbents were reused twice (Fig. 7).

The advantage of using zein-based sorbents containing laccase is that dye removal using sorbents is operationally simple. Also, incorporating laccase would allow sorbent reuse, because laccase degrades Trypan blue. Importantly, the proposed sorbents could be used to produce filters in situ, for instance around river beds or lakes polluted with dyes. Sorbents proposed in previous studies for dye removal were not applicable for in situ applications, as discussed above.

**Fig. 8:**
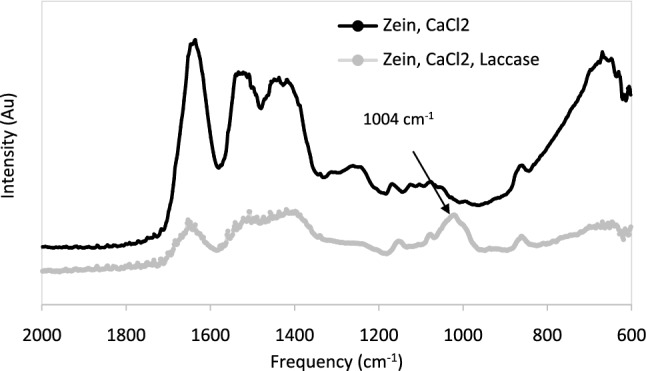
Zein sorbents coagulated with CaCl_2_ with and without laccase

It is noted that CaCl_2_ was not an effective coagulant at pH = 10. When zein sorbents were prepared at pH = 13 and effectively coagulated with CaCl_2_, laccase could be incorporated inside the sorbents. The successful incorporation of laccase in zein sorbents was verified with FT-IR, which showed a distinctive peak at 1031 cm^−1^ in the spectra of zein sorbents containing laccase (Fig. 8). This peak is characteristic of C–O–C bonds in laccase [56, 57] and was absent in zein sorbents without laccase. However, when zein sorbents were prepared at pH = 13 and coagulated with CaCl_2_, the color of the sorbent surface remained blue (instead of transitioning to purple, as was observed with free laccase). An alternative approach was thus attempted. Trypan blue was initially sorbed onto zein sorbents prepared at pH = 13 (without laccase and coagulated with CaCl_2_) and were immersed into a bath of free laccase (125 mg/L). Once again, the surface of the sorbents remained blue over a period of 11 days (rather than turning purple), indicating that enzymatic degradation of Trypan blue was inhibited. These results suggest that laccase was not able to degrade Trypan blue when it was sorbed onto zein sorbents prepared at pH = 13. This is because laccase activity is hindered at very alkaline pH [39]. As mentioned earlier and as demonstrated in our previous study, laccase activity is optimal at pH = 5, but it is nonetheless active at pH = 10 [39]. Instead, further raising the pH to pH = 13 renders laccase inactive [39].

## Conclusions

Zein powder and zein-based sorbents were used to remove Trypan blue from water at 21 °C. Part of the zein-based sorbents contained laccase, a dye-degrading enzyme. Zein-based sorbents without laccase were obtained by coagulating zein dissolved at pH = 13 with either CaCl_2_, Fe_2_Cl_3_ or citric acid, or by coagulating zein dissolved in ethanol with DI water. Zein powder was the most effective among the sorbents tested, and it sorbed over 4000 mg Trypan blue/kg sorbent (as determined through spectrophotometry). Removal efficiency was lower with coagulated zein sorbents, likely because of their lower surface area. Trypan blue removal was 500 mg Trypan blue/kg sorbent for sorbents coagulated with Fe_2_Cl_3_ and approximately 140 mg Trypan blue/kg sorbent for all other sorbents. While less effective, coagulated zein-based sorbents are easy to extract from water after being used. Future research should focus on the development of zein sorbents with high surface area, but easy to retrieve from water. The sorption isotherm of Trypan blue onto zein-based sorbents was a Type II isotherm, suggesting physisorption. Trypan blue could desorb from zein-based coagulated sorbents (without laccase) when they were immersed in DI water after sorption, but desorption was limited after 24 h. Sorbents were also produced by injecting zein solutions in geological media and subsequently solidifying them with CaCl_2_ solutions. This is the first study in which injectable sorbents were formed in situ for the removal of Trypan blue. Spectrophotometry and ESI-MS demonstrated that free laccase degraded Trypan blue in water. Laccase immobilized in zein-based sorbents prepared at pH = 10 and coagulated with Fe_2_Cl_3_ was also active and could degrade Trypan blue. Laccase-containing sorbents were therefore self-cleaning, because the dye was degraded after being absorbed. This is the first study showing the effectiveness of incorporating laccase into zein sorbents for the removal of Trypan blue.

## Supplementary information

Supplementary file1 (DOCX 510kb)

## Data Availability

The data that support the findings of this study are available from the corresponding author upon reasonable request.
