# Supplementary material for: Trypan blue removal from water with zein sorbents and laccase
Source: SN Appl Sci. 2021 Jan 7;3(1):29. doi: 10.1007/s42452-020-04107-w (PMC7790779; doi:10.1007/s42452-020-04107-w)
Supplement: Supplementary file 1 — Supplementary file1 (DOCX 510kb) [file 42452_2020_4107_MOESM1_ESM.docx]

**Supporting Information for the Manuscript “Trypan Blue Removal from Water with Zein Sorbents and Laccase”**

Tatianna Marshall^1^, Kristine Lamont^1^, Alejandro G. Marangoni^2^, Loong-Tak Lim^2^, Xiuju Wang^2^, Erica Pensini^1*^

^1^University of Guelph, School of Engineering, 50 Stone Road East, Guelph (ON), N1G 2W1, Canada

^2^University of Guelph, Food Science Department, 50 Stone Road East, Guelph (ON), N1G 2W1, Canada

*Corresponding author: email: epensini@uoguelph.ca; phone: +1 519-824-4120 ext. 56746

This supporting information file contains the calibration curve used to determine Trypan Blue concentrations in water using a Hach spectrophotometer (Fig. SI.1). This supporting information file also contains the curve showing the sorption of Trypan blue onto zein sorbents coagulated with calcium chloride, under quiescent conditions. This file further contains ESI-MS spectra of water samples containing Trypan Blue, before and after treatment with laccase (Figs. SI.3-SI.5). Finally, this file shows Trypan blue solutions after sorption of Trypan blue with sorbents containing laccase (Fig. 6).

**Figure SI.1** Calibration curve for Trypan Blue concentration determination using a Hach spectrophotometer.

**Figure SI.2** Sorption isotherm of Trypan blue onto zein coagulated with CaCl_2_ (measured after 24 hrs sorption) under quiescent conditions.


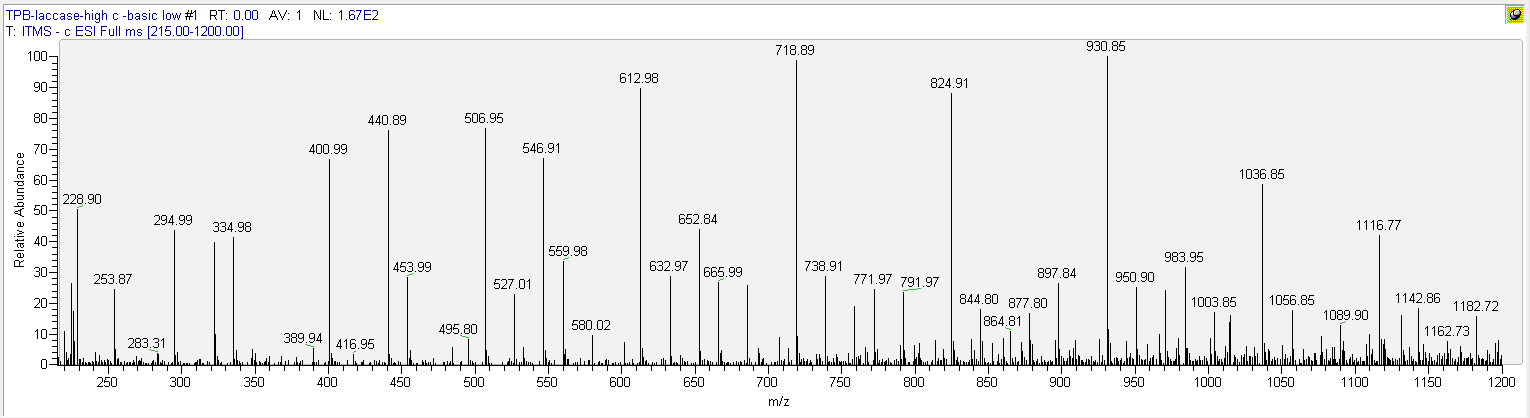

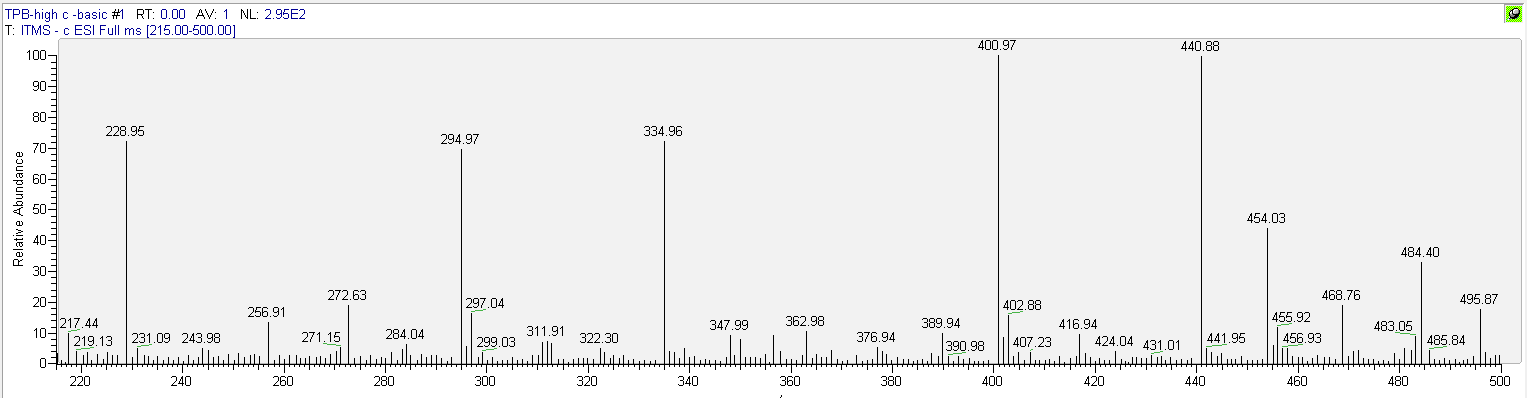


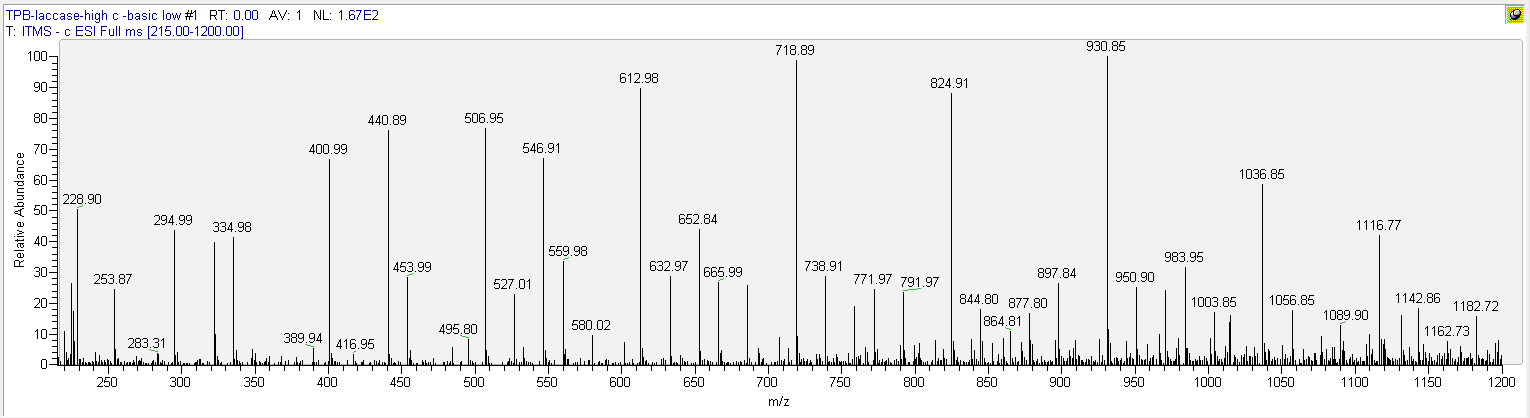


**Figure SI.3** ESI-MS spectra of water samples containing Trypan Blue before (top) and after (bottom) treatment with laccase in DI water. The pH was adjusted to pH=11 immediately before analyzing the samples.


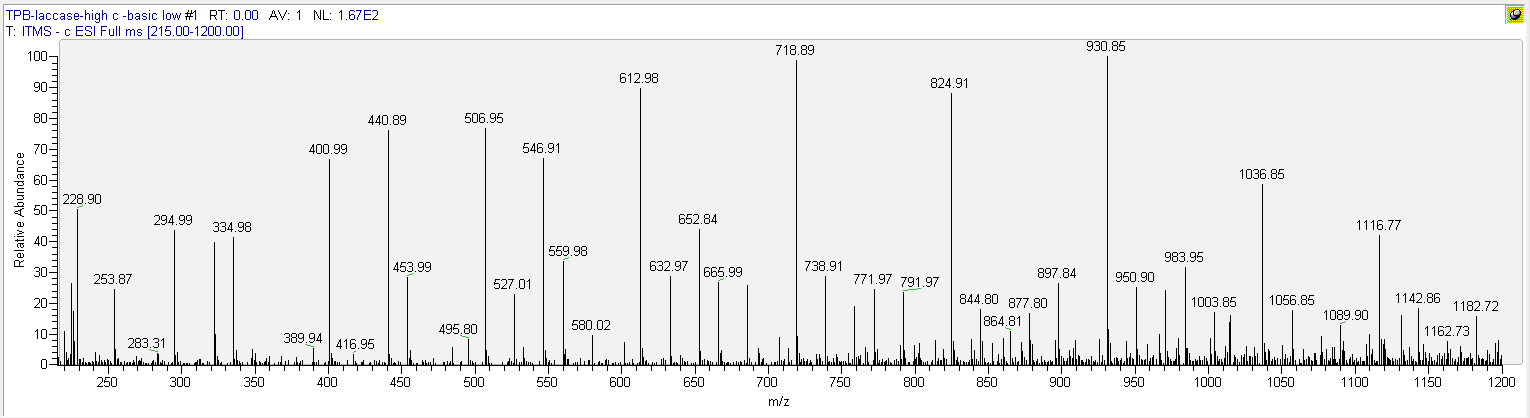

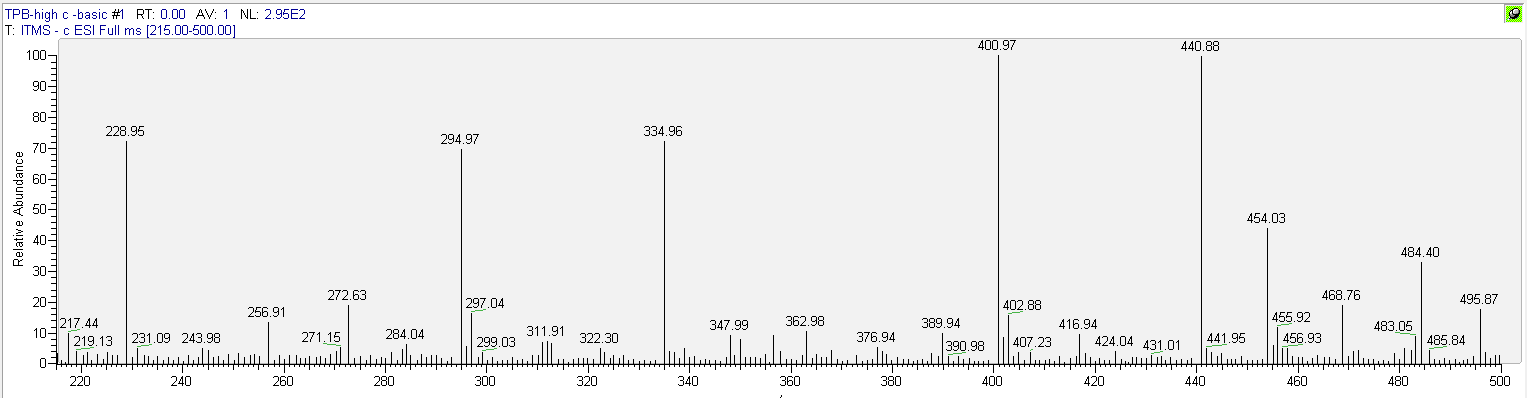


**Figure SI.4** ESI-MS spectra of water samples containing Trypan Blue before treatment with laccase in DI water. The pH was adjusted to pH=11 immediately before analyzing the samples.


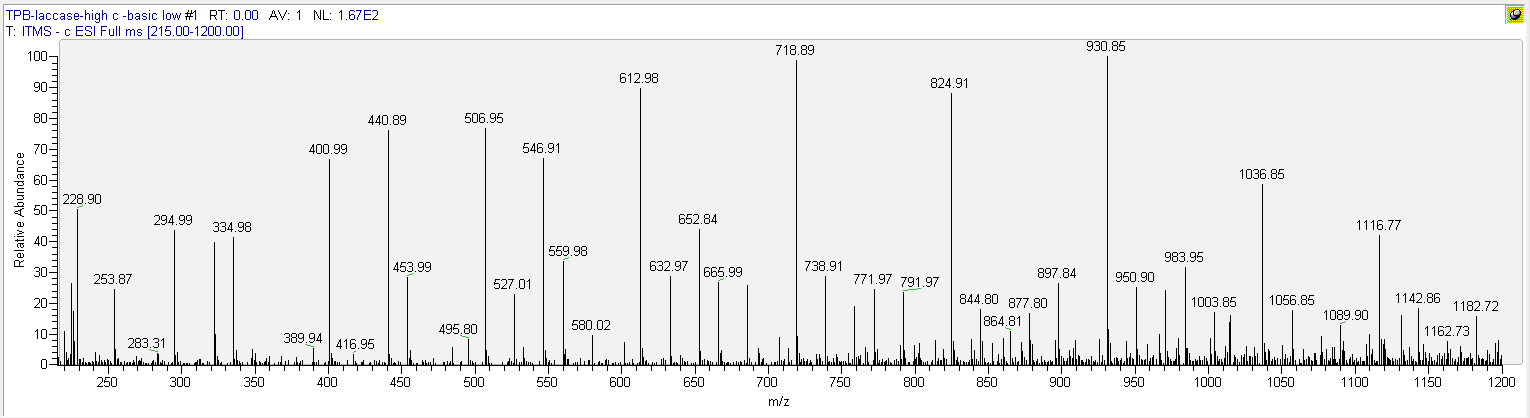

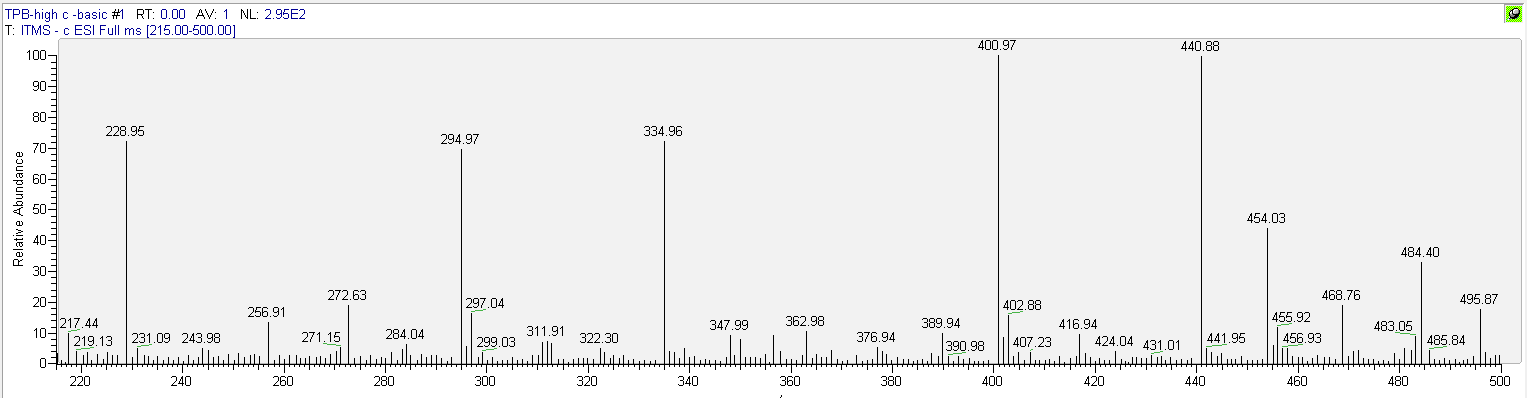

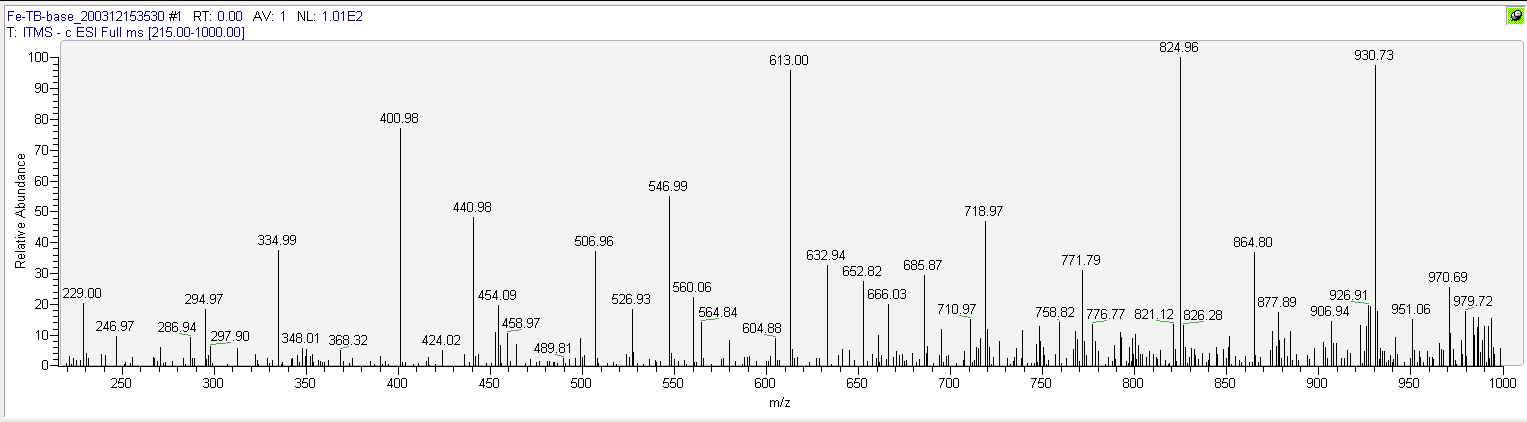


**Figure SI.5** ESI-MS spectra of water samples containing Trypan Blue before (top) and after (bottom) treatment with laccase in 0.5 M Fe_2_Cl_3_ solutions. The pH was adjusted to pH=11 immediately before analyzing the samples.


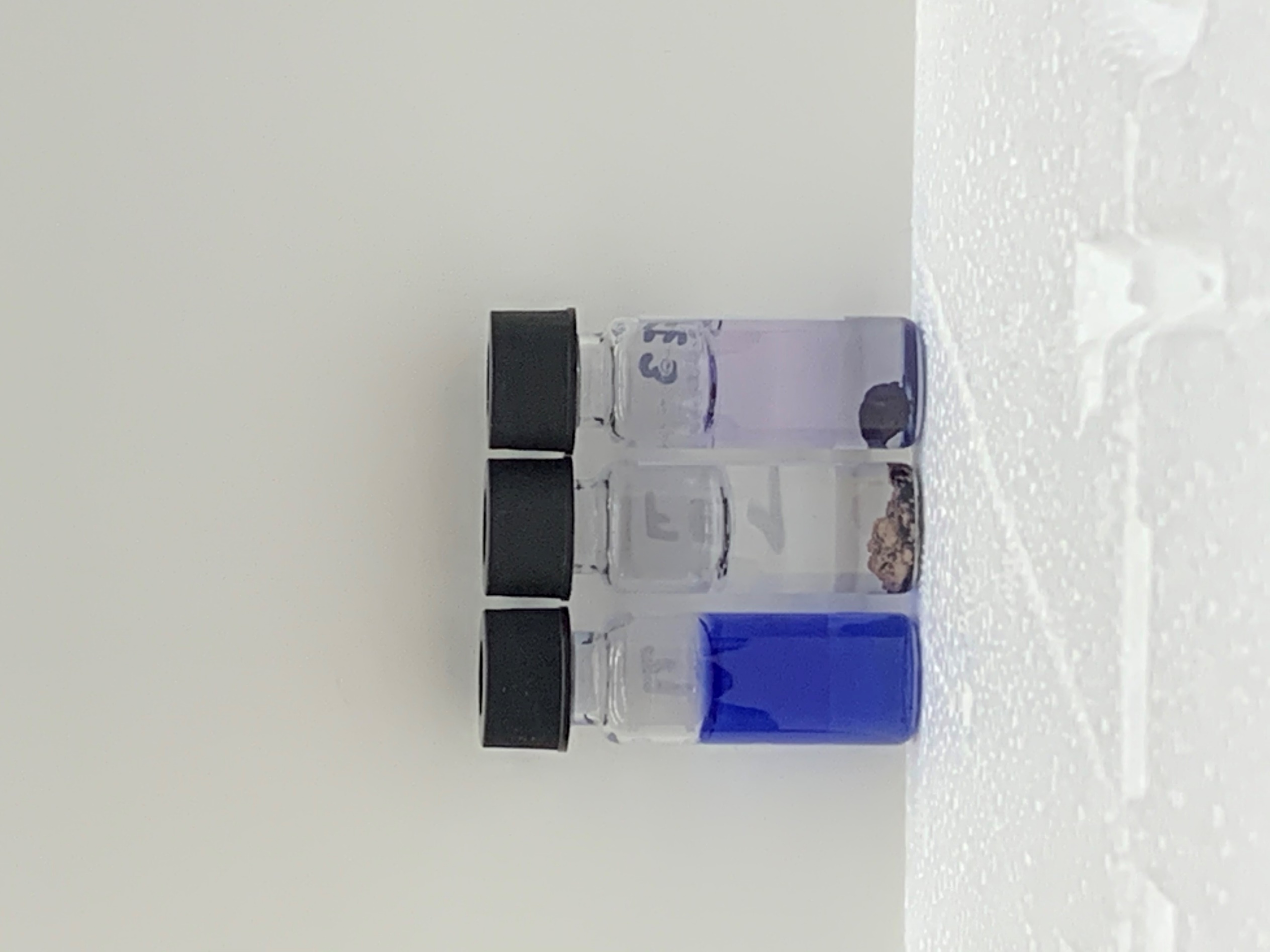


**Figure SI.6** Photograph of Trypan blue solutions before and after sorption. Left: original Trypan blue solution (19 mg/L); Centre: Trypan blue solution after sorption with a sorbent used for the first time (sorbent mass= 0.068 g); Left: Trypan blue solution after sorption with a sorbent used for the second time (sorbent mass= 0.111 g). The Trypan blue solution volume was 3 mL.
